# Sesamoid Bone Reduction in Hallux Valgus: Comparing Radiological Outcomes of Hallux Valgus Following Distal Chevron Osteotomy and Modified McBride Procedure

**DOI:** 10.3390/jcm13247590

**Published:** 2024-12-13

**Authors:** Po-Han Su, Chung-Wei Lin, Cheng-Hung Chiang, Wei-Chih Wang, Chen-Wei Yeh, Hsien-Te Chen, Yi-Chin Fong, Chien-Chung Kuo

**Affiliations:** 1 Department of Orthopedic Surgery, China Medical University Hospital, China Medical University, No. 2, Xueshi Rd., North Dist., Taichung City 404, Taiwan; minsotwkg21@hotmail.com (P.-H.S.);; 2 Department of Orthopedic Surgery, China Medical University Beigang Hospital, China Medical University, No. 123, Xinde Rd., Beigang Township, Yunlin County 651, Taiwan; 3 Department of Sports Medicine, College of Health Care, China Medical University, No. 91, Xueshi Rd., North Dist., Taichung City 404, Taiwan; 4 Spine Center, China Medical University Hospital, China Medical University, No. 2, Xueshi Rd., North Dist., Taichung City 404, Taiwan; 5 Department of Orthopedic Surgery, School of Medicine, China Medical University, No. 91, Xueshi Rd., North Dist., Taichung City 404, Taiwan

**Keywords:** hallux valgus, distal chevron osteotomy with a modified McBride procedure, sesamoid reduction

## Abstract

**Background**: Hallux valgus is a common forefoot disorder with hundreds of proposed management techniques. Distal chevron osteotomy with a modified McBride procedure has been traditionally recommended for mild hallux valgus because of its simple and less invasive nature with fewer complications, faster recovery, and reliable outcomes. In recent years, the indications for this procedure have expanded to include hallux valgus with severe deformities. This study aims to compare the radiographic outcomes of the surgical treatment for moderate versus severe hallux valgus patients from the perspective of sesamoid bone reduction. **Materials and Methods**: A retrospective review of medical records identified 99 feet from 94 patients that were treated with distal chevron osteotomy with a modified McBride procedure. These feet were divided by the preoperative hallux valgus angle and intermetatarsal angle into the moderate and severe groups. **Results**: Postoperative radiographic parameters significantly improved in both groups after treatment, indicating adequate deformity correction. Improvement in the sesamoid position was better in the moderate group compared to that in the severe group. The recurrence rate in the severe group was higher than that in the moderate group without statistical significance. **Conclusions**: Based on the radiographic results of the postoperative position following sesamoid reduction, the distal chevron osteotomy with a modified McBride procedure is effective for treating moderate hallux valgus deformities. However, this treatment strategy may be associated with a higher recurrence rate in cases of severe hallux valgus deformity. A complete reduction in the sesamoids should be emphasized during the management of severe hallux valgus deformity.

## 1. Introduction

Hallux valgus (HV) is a common forefoot disorder with a prevalence of 23% among adults aged 18–65 years and 35.7% in those over 65 years [1]. This progressive tri-planar deformity, more common in women, is characterized by valgus deviation and pronation of the big toe, varus angulation of the first metatarsal, lateral sesamoid displacement, and bunion formation at the first metatarsophalangeal (MTP) joint [2]. Hallux valgus often leads to joint incongruency and symptoms such as medial eminence pain, plantar callosities, and functional disability.

The surgical correction of hallux valgus has advanced significantly, with numerous techniques, including proximal and distal metatarsal osteotomies. These are categorized based on severity [3,4,5,6,7,8,9,10,11,12,13,14]. Mann and Coughlin classified hallux valgus into three types based on the hallux valgus angle (HVA) and intermetatarsal angle (IMA): mild (HVA < 20°, IMA < 11°), moderate (HVA 20–40°, IMA 11–16°), and severe (HVA > 40°, IMA > 16°) [15]. Proximal metatarsal osteotomies are typically recommended for severe cases due to their high corrective potential but pose challenges such as technical difficulty, prolonged recovery, and risks of hallux varus, first metatarsal shortening, and delayed union [3,10,12,13]. Recent studies suggest that distal metatarsal osteotomy with soft tissue release can effectively address severe hallux valgus with fewer complications. For example, Bai et al. reported successful correction in 94% of patients with low complication rates, while Deenik et al. and Lee et al. demonstrated comparable outcomes between distal and proximal osteotomies, favoring the less invasive distal approach for its faster recovery [4,8,9]. Cassinelli et al. illustrated a step-by-step surgical strategy of distal metatarsal osteotomy for moderate-to-severe hallux valgus and showed that the procedure could achieve and maintain the desired correction and outcome without a protracted recovery period [6].

Achieving complete and lasting correction requires thorough soft tissue release, including the adductor hallucis tendon, sesamoid suspensory ligament, and lateral MTP capsule. These steps help in properly positioning the proximal phalanx on the metatarsal head and aligning the sesamoids relative to the crista beneath the metatarsal head [16]. Failure to fully reduce the sesamoids postoperatively can increase the risk of hallux valgus recurrence [17].

Despite promising results, few studies have explored the efficacy of distal osteotomy in severe hallux valgus, particularly from the perspective of sesamoid reduction. This study aimed to evaluate radiological outcomes in moderate versus severe hallux valgus deformities, hypothesizing that greater preoperative severity correlates with residual deformity at follow-up.

## 2. Materials and Methods

### 2.1. Patient Enrollment

This study was a retrospective review of patients with symptomatic moderate-to-severe hallux valgus deformities who underwent surgical correction between January 2017 and September 2020 at a single medical center. Prior to surgery, all patients had received appropriate conservative treatment, including nonsteroidal anti-inflammatory medication, stretching exercise, activity and shoe modification, orthosis, and arch support for at least 6 months; however, their symptoms persisted. After thorough communication between the patient and the attending surgeon during outpatient visits, a mutual decision was made to proceed with surgical treatment. Postoperatively, the patient was followed up in the outpatient clinic for at least one year. During these visits, with the patient’s consent and after obtaining a signed informed consent form, relevant medical data were collected for subsequent research. To protect the patient’s privacy, the original medical data were kept confidential and were accessible only to the principal investigator and the author responsible for statistical analysis. These data were not shared with the other co-authors. Upon the completion of the study, all research data were destroyed and were not used for any additional studies. The study adhered to the principles of the Declaration of Helsinki and was approved by the Research Ethics Committee of China Medical University Hospital, Taichung, Taiwan (protocol ID: CMUH109-REC2-021, approval date: 2020/04/07). During the study period, patient data and personal information were carefully safeguarded and were entirely deleted upon the completion of this study. Hallux valgus severity was classified as moderate (HVA 20–40°, IMA 11–16°) or severe (HVA > 40°, IMA > 16°) [15,18].

**Figure 1:**
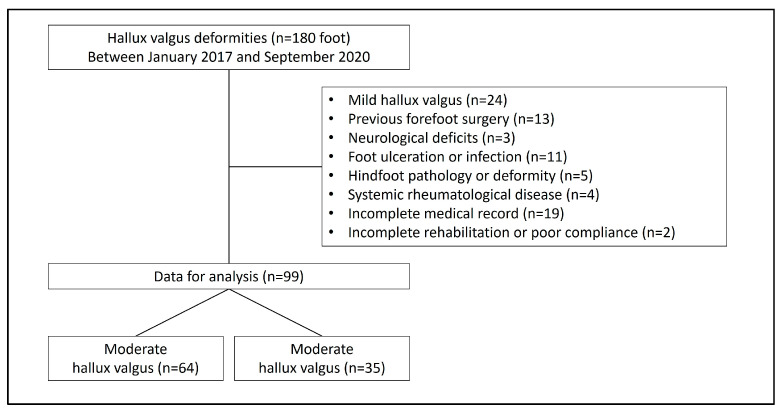
Participant enrollment flow chart.

The inclusion criteria included patients aged from 18 to 80 years who underwent treatment for hallux valgus deformity with persistent symptoms despite adequate conservative management between January 2017 and September 2020. All patients were treated using distal chevron osteotomy and the modified McBride procedure, with a postoperative follow-up of at least one year. A total of 180 affected feet were initially included for surgical treatment. The exclusion criteria included mild hallux valgus, any history of surgical intervention on the forefoot regardless of indication, neurological deficits, foot ulceration or infection (including diabetic foot or peripheral arterial occlusive disease), concurrent hindfoot pathology or deformity, systemic rheumatological disease, incomplete medical records (such as missing clinical or radiographic data or follow-up less than 12 months), and inability to comply with postoperative assistive devices or rehabilitation. After applying these criteria, 81 affected feet were excluded, leaving 99 affected feet for data analysis (Figure 1).

A total of 99 feet were divided into moderate (M, 64 feet with a preoperative HVA of 20–40° and IMA of 11–16°) and severe (S, 35 feet with a preoperative HVA > 40° and IMA > 16°) groups, according to preoperative HVA and IMA.

### 2.2. Radiographic Evaluation

All radiographs were obtained using the same protocol from a single medical center. Anteroposterior (AP) and oblique weight-bearing radiography were performed preoperatively and during each follow-up. AP and oblique non-weight-bearing radiographs were obtained immediately after surgery. An observer, who did not participate in the operative team and was blinded to the surgical outcome, performed the radiographic measurements.

HVA was defined as the angle between the longitudinal axis of the first metatarsal and that of the first proximal phalanx. IMA was defined as the angle between the longitudinal axis of the first and second metatarsals. The longitudinal axis of the first and second metatarsals was defined as the line connecting the center of the metatarsal head and the proximal articular surface of the metatarsal, while the longitudinal axis of the proximal phalanx corresponded to a line connecting the centers of the proximal and distal ends of the diaphysis [19]. The distal metatarsal articular angle (DMAA) was determined as the angle between a line perpendicular to the longitudinal axis of the first metatarsal and a line parallel to the distal articular surface of the metatarsal head [20].

**Figure 2:**
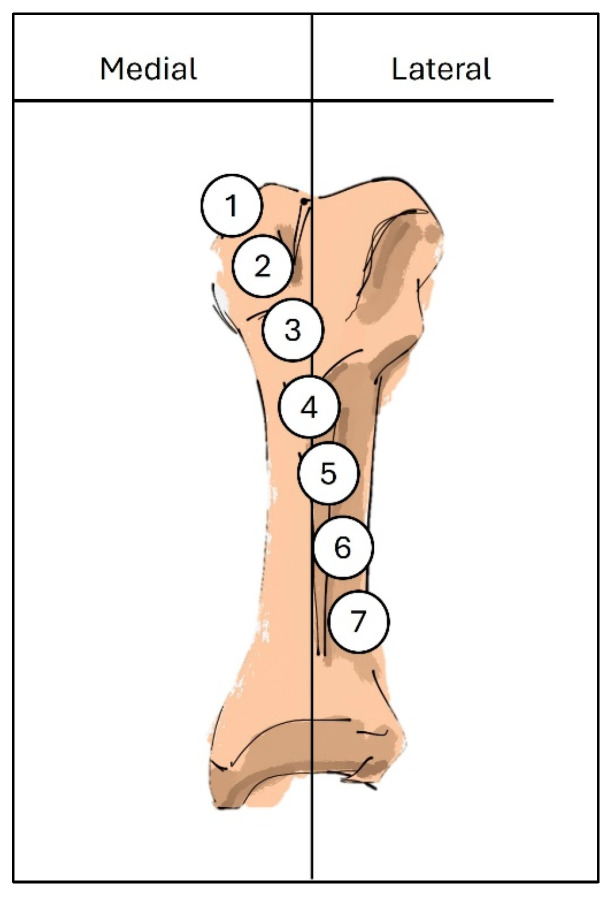
The illustration demonstrates how the severity of sesamoid subluxation or dislocation can be assessed using the grading system developed by Hardy and Clapham. The medial-to-lateral position of the medial sesamoid (indicated by numbered circles) is evaluated in relation to the longitudinal axis of the first metatarsal (depicted by the vertical line).

The sesamoid, in relation to the first metatarsal head, was measured by relating the medial sesamoid to the longitudinal axis of the first metatarsal, and graded from 1 to 7, according to the classification by Hardy and Clapham (Figure 2) [21]. The congruency of the first MTP joint was also documented. The joint congruency of the first metatarsophalangeal (MTP) joint was assessed by determining whether two lines, representing the base of the proximal phalanx and the estimated cartilage surface of the first metatarsal head, were parallel [22,23].

The recurrence of hallux valgus deformity was defined as an HVA ≥ 20° on weight-bearing radiographs [11,17,24,25].

### 2.3. Surgical Technique

The surgery consisted of a modified McBride procedure, varus traction, and a distal metatarsal chevron osteotomy. All surgeries, as well as preoperative planning and clinical follow-ups, were conducted by the chief orthopedic surgeon, who specialized in foot and ankle surgery.

During surgery, the patients were placed in the supine position with a tourniquet applied to their thighs. Subsequently, spinal anesthesia was induced. The procedure began with a 1.5 cm skin incision over the first web space. The adductor tendon and deep transverse metatarsal ligament were released from distal to proximal, which was considered the most important part as it allowed lateral shifting of the metatarsal capital fragment and subsequent improvement in sesamoid reduction. Forced varus traction was then applied to the hallux, with a recognized pop in most cases.

**Figure 3:**
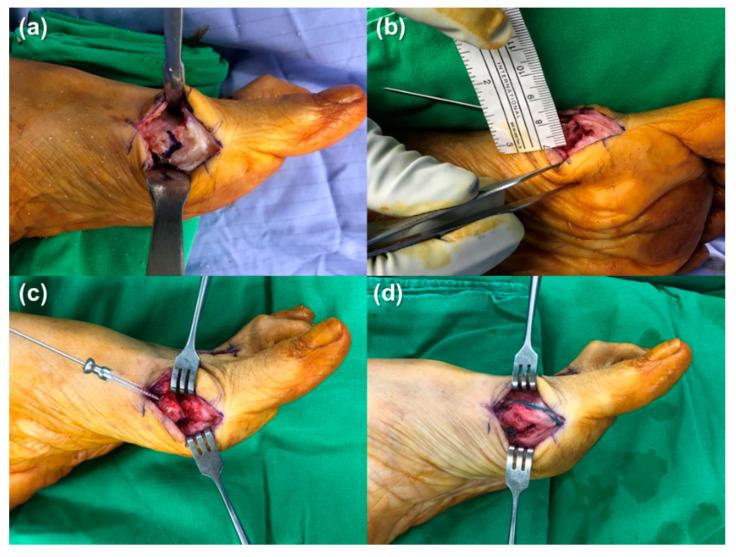
(**a**) A “V”-shaped osteotomy (Chevron osteotomy) of the distal metatarsal was planned. The center of the metatarsal head is approximately 1 cm proximal to the joint. (**b**) The first metatarsal head was shifted laterally with a usual displacement of approximately 5–7 mm. (**c**) A bioabsorbable screw was placed into the capital fragment along the guidewire for fixation. (**d**) Nonabsorbable no. 2 mattress-type sutures were placed in the base of the capsular flap and pulled through. After placing the hallux in supination, extension, and medial abduction position, the sutures were tightened.

A 5 cm skin incision was centered over the medial eminence. The medial capsule was split vertically from distal to proximal. A full-thickness flap, including skin and the medial capsule, was elevated to expose the medial eminence. Using an oscillating saw, the medial eminence was removed in line with the medial border of the first metatarsal. Care was taken to avoid causing a notch in the proximal metatarsal. The center of the metatarsal head was marked, and a distal chevron osteotomy was performed at a 60° angle (Figure 2). Osteotomy was performed perpendicular to the foot axis to maintain the metatarsal length. The distal metatarsal capital fragment was then translated 5–7 mm laterally to accomplish the correction (Figure 2). A Kirschner wire with a 1.2 mm diameter was then inserted through the osteotomy site, from the proximal medial dorsal to distal lateral plantar, into the metatarsal head for provisional fixation. Intraoperative C-arm fluoroscopy was performed to confirm displacement and sesamoid repositioning under the metatarsal head. The osteotomy site was stabilized with a Kirschner wire or bioabsorbable screw (Figure 3). The medial overhanging ledge of the bone was smoothened using an oscillating saw. Irrigation was performed to flush away bone dust.

**Figure 4:**
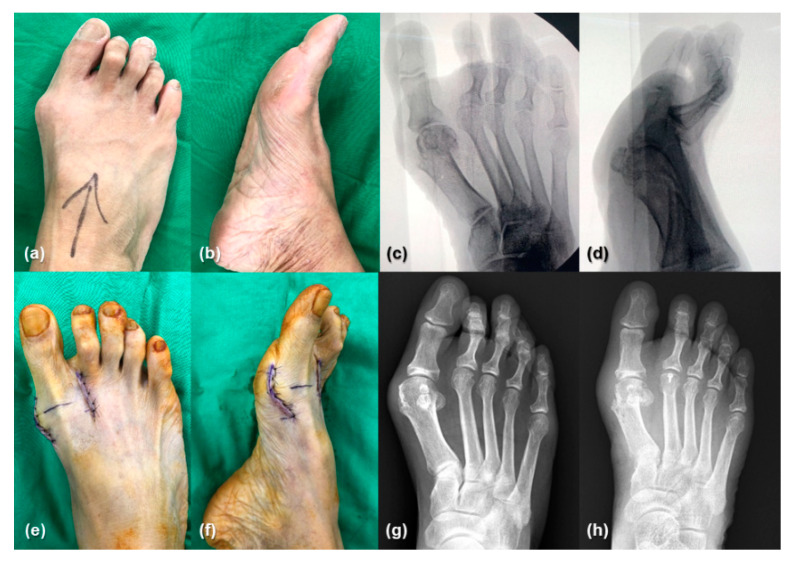
A 61-year-old woman presented with symptomatic hallux valgus. (**a**,**b**) Preoperative gross pictures depict hallux valgus deformity. (**c**) Anteroposterior and (**d**) lateral intraoperative C-arm fluoroscopy images depict restored alignment. (**e**,**f**) Postoperative gross pictures depict proper deformity correction. (**g**) A preoperative radiograph depicts an HVA of 38° and IMA of 15°. (**h**) A radiograph at 1 month after surgery depicts an HVA of 12° and IMA of 6°.

A 1.6 mm Kirschner wire was used to create a dorsal-to-plantar hole in the metatarsal proximal to the osteotomy site. Nonabsorbable no. 2 mattress-type sutures were placed at the base of the capsular flap and pulled through the previous hole in the metatarsal (Figure 2). The sutures were subsequently tightened, with the hallux gently placed in a supinated, extended, and medially abducted position. C-arm fluoroscopy was used to verify the hallux position without excessive varus (Figure 4). Furthermore, the deep capsule was closed with an absorbable no. 1 suture and the wound was closed in layers. Tape was used over the interphalangeal (IP) joint and down to the proximal metatarsal of the hallux to maintain reduction. A gauze roll was placed between the first and second toes to maintain the positioning of the greater toe. Finally, the foot was wrapped with an elastic bandage.

### 2.4. Postoperative Care

A short leg splint was applied postoperatively. Patients were instructed to avoid weight-bearing during the first postoperative day. Weight-bearing on the heel was permitted only after wearing a cast shoe or forefoot offloading shoe. During the first two weeks, patients were advised to reduce weight-bearing on the surgical foot, limited to the heel with the use of a cast shoe, while the contralateral foot bore the remaining weight. Gradual progression to full weight-bearing on the heel of the surgical foot was recommended over the following four weeks. The postoperative protocol for forefoot included one month of non-weight-bearing, followed by two months of reduced weight-bearing with orthotic protection. Gentle range-of-motion exercises for the big toe were initiated one week after surgery. Patients were encouraged to wear shoes with arch support and to use a nighttime bunion splint for up to three months to reduce tension on the medial skin. Starting three months postoperatively, patients were encouraged to wear shoes with arch support to reduce pressure beneath the hallux and the first metatarsal head [26].

### 2.5. Statistical Analysis

Statistical analyses were performed using SPSS ver. 19.0 (SPSS Inc., Chicago, IL, USA). The differences in the measured variables between the two groups were analyzed using the Mann–Whitney U test. Fisher’s exact test was used to compare dichotomous data. The Wilcoxon signed-rank test was used to compare the preoperative and postoperative radiographic results of both groups. A *p*-value < 0.05 was considered statistically significant.

## 3. Results

Ninety-four patients (99 feet) were included in the study. Of these, 78 were women and 16 were men. A total of 62 patients (64 feet) were allocated to group M, while 32 (35 feet) were assigned to group S. Baseline patient characteristics are summarized in Table 1. No significant differences in age, sex, body mass index, operation time, and follow-up duration were observed between the two groups.

In group M, the average HVA, IMA, and DMAA improved from 31.5 ± 4.81° to 7.25 ± 3.76° (*p* < 0.001), from 12.7 ± 2.42° to 5.77 ± 1.97° (*p* < 0.001), and from 12.7 ± 4.35° to 4.7 ± 2.38° (*p* < 0.001), respectively. In group S, the average HVA, IMA, and DMAA improved from 41.9 ± 6.6° to 8.19 ± 3.95° (*p* < 0.001), from 16.47 ± 2.83° to 6.5 ± 2.16° (*p* < 0.001), and from 15.8 ± 6.34° to 5.66 ± 2.87° (*p* < 0.001), respectively. All patients had preoperative incongruity in the first MTP joint. The congruency of the first MTP joint was restored in 100% of patients in group M and 97.1% of patients in group S. Joint congruency in both groups was significantly different between preoperative and postoperative conditions (*p* < 0.001). The average preoperative medial sesamoid position was 4.86 ± 1.11 and 5.66 ± 1.06 in groups M and S, respectively, while their postoperative counterparts were 2.89 ± 1.16 and 3.71 ± 1.32 in groups M and S, respectively. The medial sesamoid position improved significantly in both groups after surgery (*p* < 0.001, Table 2).

Regarding the postoperative radiographic parameters summarized in Table 3, the immediate postoperative HVA, IMA, and DMAA were larger in group S than those in group M, with no statistical significance. All 99 feet had an HVA < 15° and IMA < 10° immediately after surgery. In terms of deformity correction, the correction angles of the HVA and IMA in group S were significantly larger than those in group M (*p* < 0.001). The correction angle of DMAA in group S was slightly higher than that in group M, with no statistical significance (*p* = 0.4). At the time of the most recent follow-up, HVA and IMA in group S were greater than those in group M, with no statistical significance (*p* = 0.052 and *p* = 0.107, respectively). The loss of correction angle of HVA and IMA did not differ significantly between the two groups (*p* = 0.374 and *p* = 0.392, respectively). However, a significant difference in the sesamoid position after surgery between the two groups was observed (*p* = 0.004). Recurrence of hallux valgus deformity (HVA ≥ 20°) was observed in three feet (4.69%) in group M and four feet (11.43%) in group S. The prevalence of recurrence in group S was higher than that in group M, with no statistical significance (*p* = 0.24).

No intraoperative fractures, infections, wound healing problems, fragment displacement, nonunion, and delayed union were encountered in our patients. Additionally, there was no persistent pain, painful transfer metatarsalgia, or complex regional pain syndrome.

## 4. Discussion

### 4.1. Correction of Hallux Valgus with Distal Osteotomy and Soft Tissue Procedure

Approximately over 100 procedures have been proposed for the treatment of hallux valgus deformities. The goal of operative management is to alleviate pain and improve functional ability in patients refractory to conservative treatment. Historically, proximal metatarsal osteotomy has been chosen as the main procedure for severe hallux valgus deformity due to its high corrective ability. However, potential barriers to this procedure include highly demanding surgical techniques, longer postoperative recovery time, and increased complication risks [2,7,10,27,28]. Distal metatarsal osteotomy was originally proposed for mild-to-moderate hallux valgus and previously considered inadequate for severe hallux valgus management because of its limited degree of possible correction. To overcome this disadvantage, some surgeons have added a distal soft tissue procedure for cases with more severe deformities [29,30,31,32]. Chen et al. described the technique of combining intra-articular lateral soft tissue release with distal chevron osteotomy for the treatment of moderate-to-severe hallux valgus, with satisfactory outcomes and a low incidence of avascular necrosis [29]. Peterson et al. also reported significant HVA and IMA corrections and an 84% satisfaction rate with a similar method [30]. Another study further revealed that the addition of a soft tissue procedure facilitated the lateral displacement of the metatarsal head and allowed the correction of the deformity beyond the usual 30° of HVA and 15° of IMA [32]. Recently, the procedure has become even more popular due to its ease of operation, with a shorter incision and fewer complications, as well as its reliability for hallux valgus deformity correction, which has been validated in several reports [4,6,7,8,29,30,31,32].

This study evaluated the efficacy of distal metatarsal osteotomy with a modified McBride procedure for moderate-to-severe hallux valgus deformities. We compared radiographic measurements obtained preoperatively, postoperatively, and at most recent follow-up. Parameters, including HVA, IMA, DMAA, joint congruency, and sesamoid position, were evaluated. Overall, the radiographic parameters showed significant improvement after surgery. Regarding hallux valgus assessment, radiographically measured HVA and IMA were more reliable than DMAA [33]. In both groups, the mean HVA and IMA normalized to <15° and <9°, respectively, indicating adequate deformity correction. Similarly, the decrease in DMAA corresponded to an improvement in joint congruency in both groups.

### 4.2. Recurrence Rate and Reduction of Sesamoid

The correction of sesamoid subluxation has been an important surgical goal, as it removes the dynamic deformation force originating from the flexor hallucis brevis [34]. The incomplete reduction in the sesamoid could lead to postoperative complications, including hallux valgus recurrence, sesamoid irritation, and altered walking mechanics [17,35]. Groups M and S exhibited significant sesamoid reduction postoperatively. However, the improvement in the sesamoid position in group M was greater than that in group S.

The recurrence of hallux valgus deformity has been commonly described in the literature, with a range of 8–25% [17,36,37,38,39,40]. However, different definitions of hallux valgus recurrence exist. Veri et al. defined recurrence as an increase in HVA ≥ 10° [41], while Clarke et al. defined recurrence as HVA > 15°. Coughlin et al., Okuda et al., and several other studies have defined recurrence as HVA > 20° [11,17,24,25]. Since the lower HVA threshold for moderate-to-severe hallux valgus is 20°, HVA > 20° was defined as recurrence in this study. The causes of recurrent hallux valgus are multifactorial, including patient-related and surgical factors. Patient-related factors include preoperative radiographic parameters, medical comorbidities, the continuous use of high-heeled shoes, smoking, and compliance with postoperative instructions. Surgical factors include choice of appropriate procedure, technical competency, and undercorrection. Park et al. investigated the relationship between radiographic parameters and recurrence, and found that a preoperative HVA ≥ 40°, preoperative metatarsus adductus angle ≥ 23°, immediate postoperative HVA ≥ 8°, and immediate postoperative sesamoid position of grade 4 or greater were significantly associated with recurrence [39]. The sesamoid position has also been implicated as a recurrence risk factor. Okuda et al. revealed that incomplete postoperative sesamoid reduction could be a recurrence risk factor for hallux valgus [17].

### 4.3. Prevention of Recurrence of Severe Hallux Valgus

In this study, the recurrence rate in group S was higher than that in group M, without statistical significance (*p* = 0.24). The immediate postoperative HVA and IMA in groups M and S did not show significant differences and were within the acceptable range. However, the immediate postoperative sesamoid position in group S was worse than that in group M. The residual lateral displacement of the sesamoid might be closely related to the progression or recurrence of hallux valgus, as previously reported [17]. This result suggested that following hallux valgus deformity correction, it would be beneficial to confirm the reduction in sesamoid intraoperatively, particularly in patients with severe hallux valgus deformity. Adequate soft tissue release during the modified McBride procedure is an important part of hallux valgus surgery to ensure proper sesamoid position. However, successful lateral release is highly dependent on the surgeon’s experience. There is no current consensus on how this procedure should be performed. If inadequate sesamoid reduction is noted, additional lateral release of the dorsolateral aspect of the MTP joint capsule, sectioning of the metatarso-sesamoid suspensory ligament, and sectioning of the phalangeal insertional band might be required [17,42]. Transecting the lateral metatarsosesamoid suspensory ligament is crucial for effective lateral release in hallux valgus surgery. Preserving the lateral short sesamoid–phalangeal ligament and plantar articular capsule prevents joint instability. For severe hallux valgus, after completing the lateral release of the lateral metatarso-sesamoid suspensory ligament following the incision of the lateral articular capsule, medial capsular reefing should be performed to realign the metatarso-sesamoid complex. It is crucial to verify proper reduction and alignment by forcing the toe into overcorrection [43]. This step is considered a key factor in effectively minimizing the risk of recurrence, as it may help reduce postoperative deforming forces by releasing the contracted structures [43]. The proper bony realignment of the first metatarsal, adequate lateral displacement of the metatarsal head during a distal chevron osteotomy, adequate lateral soft tissue release, and appropriate medial capsulorrhaphy contribute to successful hallux valgus deformity correction.

### 4.4. Limitations

Our study had several limitations. First, it was a retrospective study with a relatively small number of patients. However, the baseline characteristics between the two groups were generally aligned, and we found significant improvements in radiographic parameters preoperatively and postoperatively for both groups. These findings would provide important decision-making information for preoperative severity-based treatment selection for hallux valgus deformity. The current study showed that adequate radiographic correction can be achieved for severe hallux valgus deformities with this simple and reliable procedure.

Nonetheless, research on the biomechanical factors contributing to sesamoid pronation deformity remains limited. Based on the findings of this study, further investigations are warranted to explore the forces acting on and around the sesamoid that lead to deformity. Additionally, understanding how surgical interventions can modify these mechanical forces is essential for optimizing patient outcomes.

Second, follow-up duration was relatively short (with a minimum of 1 year). However, previous studies demonstrated that there was no significant change with time [41,44]. Lastly, objective scores were not included in this study, which slightly impaired the analysis of clinical information of our patients. Given this limitation, we focused on radiological outcomes between the two groups with moderate vs. severe hallux valgus deformities. Symptoms, such as persistent pain or metatarsalgia, would be recognized as complications, if identified.

## 5. Conclusions

Based on our findings, the radiographic results of postoperative sesamoid reduction suggest that distal chevron osteotomy combined with a modified McBride procedure is both effective and reliable for treating moderate hallux valgus deformity. However, the recurrence rate was slightly higher in patients with severe hallux valgus.

We believe that complete sesamoid reduction should be further emphasized in severe cases, as it may help alleviate postoperative deforming forces by releasing contracted structures. This can be accomplished through the release of the contracted lateral soft tissue structures, tightening of the weakened medial structures, and realignment of the first metatarsal head onto the sesamoid complex. Subsequently, alignment must be verified by forcing the toe into overcorrection, which serves as a critical step in minimizing the risk of recurrence.

Nevertheless, the complex interplay of forces contributing to sesamoid deformity requires further investigation to better understand the mechanical factors involved and how surgical interventions can modify these vectors.

## Figures and Tables

**Table 1:** Patients’ demographic data.

	Group M	Group S	*p*-Value
**Sex**			
Male	13 (20.97%)	3 (9.38%)	0.442
Female	49 (79.03%)	29 (90.62%)	
**Age (years)**	52.9 ± 15.6	50.8 ± 17.3	0.666
**BMI (kg/m^2^)**	24.2 ± 4.3	23.9 ± 4.1	0.956
**Operation time (minutes)**	83 ± 25	77.3 ± 18.4	0.457
**Follow-up (months)**	20.11 ± 10.48	20.57 ± 10.23	0.727

Data are presented either as absolute numbers and percentages (%) or means ± standard deviations. BMI, body mass index.

**Table 2:** Comparison of preoperative and postoperative radiographic parameters.

	Preoperative	Postoperative	*p*-Value
**HVA (°)**			
group M	31.5 ± 4.81	7.25 ± 3.76	<0.001 *
group S	41.9 ± 6.6	8.19 ± 3.95	<0.001 *
**IMA (°)**			
group M	12.7 ± 2.42	5.77 ± 1.97	<0.001 *
group S	16.47 ± 2.83	6.5 ± 2.16	<0.001 *
**DMAA (°)**			
group M	12.7 ± 4.35	4.7 ± 2.38	<0.001 *
group S	15.8 ± 6.34	5.66 ± 2.87	<0.001 *
**Congruent joint (%)**			
group M	0 (0%)	64 (100%)	<0.001 *
group S	0 (0%)	34 (97.1%)	<0.001 *
**Sesamoid position** **(Hardy–Clapham scale)**			
group M	4.86 ± 1.11	2.89 ± 1.16	<0.001 *
group S	5.66 ± 1.06	3.71 ± 1.32	<0.001 *

Data are presented either as absolute numbers and percentages (%) or means ± standard deviations. DMAA, distal metatarsal articular angle; HVA, hallux valgus angle; IMA, intermetatarsal angle. * *p* < 0.05.

**Table 3:** Postoperative radiographic parameters of groups M and S.

	Group M	Group S	*p*-Value
**HVA (°)**			
Immediate postoperative	7.25 ± 3.76	8.19 ± 3.95	0.064
Correction angle	24.25 ± 4.89	33.68 ± 5.86	<0.001 *
Most recent follow-up	16 ± 6	18.93 ± 6.91	0.052
Loss of correction angle	8.27 ± 5.77	9.74 ± 4.79	0.374
**IMA (°)**			
Immediate postoperative	5.77 ± 1.97	6.5 ± 2.16	0.263
Correction angle	6.95 ± 2.34	9.96 ± 2.45	<0.001 *
Most recent follow-up	6.84 ± 2.22	7.96 ± 3.07	0.107
Loss of correction angle	1.08 ± 1.87	1.47 ± 2.11	0.392
**DMAA (°)**			
Immediate postoperative	4.7 ± 2.38	5.66 ± 2.11	0.342
Correction angle	8 ± 3.83	10.14 ± 4.95	0.4
**Congruent joint (%)**	100%	97.1%	0.176
**Sesamoid position** **(Hardy–Clapham scale)**			
Immediate postoperative	2.89 ± 1.16	3.71 ± 1.32	0.004 *
**Recurrence (HVA** ≥ **20°)**	3/64(4.69%)	4/35(11.43%)	0.24

Data are presented either as absolute numbers and percentages (%) or means ± standard deviations. DMAA, distal metatarsal articular angle; HVA, hallux valgus angle; IMA, intermetatarsal angle. * *p* < 0.05.

## Data Availability

The datasets used and analyzed during the current study are available from the corresponding author upon reasonable request.

## References

[1] Nix S., Smith M., Vicenzino B. (2010). Prevalence of hallux valgus in the general population: A systematic review and meta-analysis. J. Foot Ankle Res..

[2] Badekas A., Georgiannos D., Lampridis V., Bisbinas I. (2013). Proximal opening wedge metatarsal osteotomy for correction of moderate to severe hallux valgus deformity using a locking plate. Int. Orthop..

[3] Adam S.P., Choung S.C., Gu Y., O’Malley M.J. (2011). Outcomes after scarf osteotomy for treatment of adult hallux valgus deformity. Clin. Orthop. Relat. Res..

[4] Bai L.B., Lee K.B., Seo C.Y., Song E.K., Yoon T.R. (2010). Distal chevron osteotomy with distal soft tissue procedure for moderate to severe hallux valgus deformity. Foot Ankle Int..

[5] Buciuto R. (2014). Prospective randomized study of chevron osteotomy versus Mitchell’s osteotomy in hallux valgus. Foot Ankle Int..

[6] Cassinelli S.J., Herman R., Harris T.G. (2016). Distal Metatarsal Osteotomy for Moderate to Severe Hallux Valgus. Foot Ankle Int..

[7] Chuckpaiwong B. (2012). Comparing proximal and distal metatarsal osteotomy for moderate to severe hallux valgus. Int. Orthop..

[8] Deenik A., van Mameren H., de Visser E., de Waal Malefijt M., Draijer F., de Bie R. (2008). Equivalent correction in scarf and chevron osteotomy in moderate and severe hallux valgus: A randomized controlled trial. Foot Ankle Int..

[9] Lee K.B., Cho N.Y., Park H.W., Seon J.K., Lee S.H. (2015). A comparison of proximal and distal Chevron osteotomy, both with lateral soft-tissue release, for moderate to severe hallux valgus in patients undergoing simultaneous bilateral correction. Bone Jt. J..

[10] Lenz C.G., Niehaus R., Knych I., Eid K., Borbas P. (2021). Scarf osteotomy for hallux valgus deformity: Radiological outcome, metatarsal length and early complications in 118 feet. Foot Ankle Surg..

[11] Park C.H., Jang J.H., Lee S.H., Lee W.C. (2013). A comparison of proximal and distal chevron osteotomy for the correction of moderate hallux valgus deformity. Bone Jt. J..

[12] Robinson A.H., Bhatia M., Eaton C., Bishop L. (2009). Prospective comparative study of the scarf and Ludloff osteotomies in the treatment of hallux valgus. Foot Ankle Int..

[13] Saragas N.P. (2009). Proximal opening-wedge osteotomy of the first metatarsal for hallux valgus using a low profile plate. Foot Ankle Int..

[14] Saro C., Andren B., Wildemyr Z., Fellander-Tsai L. (2007). Outcome after distal metatarsal osteotomy for hallux valgus: A prospective randomized controlled trial of two methods. Foot Ankle Int..

[15] Coughlin M.J., Mann R.A., Saltzman C.L. (2007). Surgery of the Foot and Ankle.

[16] Park Y.B., Lee K.B., Kim S.K., Seon J.K., Lee J.Y. (2013). Comparison of distal soft-tissue procedures combined with a distal chevron osteotomy for moderate to severe hallux valgus: First web-space versus transarticular approach. J. Bone Jt. Surg. Am..

[17] Okuda R., Kinoshita M., Yasuda T., Jotoku T., Kitano N., Shima H. (2009). Postoperative incomplete reduction of the sesamoids as a risk factor for recurrence of hallux valgus. J. Bone Jt. Surg. Am..

[18] Ray J.J., Friedmann A.J., Hanselman A.E., Vaida J., Dayton P.D., Hatch D.J., Smith B., Santrock R.D. (2019). Hallux Valgus. Foot Ankle Orthop..

[19] Shima H., Okuda R., Yasuda T., Jotoku T., Kitano N., Kinoshita M. (2009). Radiographic measurements in patients with hallux valgus before and after proximal crescentic osteotomy. J. Bone Jt. Surg. Am..

[20] Richardson E.G., Graves S.C., McClure J.T., Boone R.T. (1993). First metatarsal head-shaft angle: A method of determination. Foot Ankle.

[21] Hardy R.H., Clapham J.C. (1951). Observations on hallux valgus; based on a controlled series. J. Bone Jt. Surg. Br..

[22] Fuhrmann R.A., Zollinger-Kies H., Kundert H.P. (2010). Mid-term results of Scarf osteotomy in hallux valgus. Int. Orthop..

[23] Li Y., Tao X., Tang K. (2022). Radiographic evaluation of congruency of the first metatarsophalangeal joint in hallux valgus. J. Orthop. Surg. Res..

[24] Bock P., Kluger R., Kristen K.H., Mittlbock M., Schuh R., Trnka H.J. (2015). The Scarf Osteotomy with Minimally Invasive Lateral Release for Treatment of Hallux Valgus Deformity: Intermediate and Long-Term Results. J. Bone Jt. Surg. Am..

[25] Coughlin M.J., Jones C.P. (2007). Hallux valgus and first ray mobility. A prospective study. J. Bone Jt. Surg. Am..

[26] Farzadi M., Safaeepour Z., Mousavi M.E., Saeedi H. (2015). Effect of medial arch support foot orthosis on plantar pressure distribution in females with mild-to-moderate hallux valgus after one month of follow-up. Prosthet. Orthot. Int..

[27] Clarke T.A.C., Platt S.R. (2021). Treatment of hallux valgus by Scarf osteotomy-rates and reasons for recurrence and rates of avascular necrosis: A systematic review. Foot Ankle Surg..

[28] Sammarco G.J., Idusuyi O.B. (2001). Complications after surgery of the hallux. Clin. Orthop. Relat. Res..

[29] Chen Y.J., Hsu R.W., Shih H.N., Huang T.J., Hsu K.Y. (1996). Distal chevron osteotomy with intra-articular lateral soft-tissue release for treatment of moderate to severe hallux valgus deformity. J. Formos. Med. Assoc..

[30] Peterson D.A., Zilberfarb J.L., Greene M.A., Colgrove R.C. (1994). Avascular necrosis of the first metatarsal head: Incidence in distal osteotomy combined with lateral soft tissue release. Foot Ankle Int..

[31] Selner A.J., Ginex S.L., Selner M.D. (1994). Tricorrectional bunionectomy for correction of high intermetatarsal angles. J. Am. Podiatr. Med. Assoc..

[32] Steinböck G. (2003). Chevron-osteotomy for the treatment of Hallux valgus. Foot Ankle Surg..

[33] Coughlin M.J., Freund E. (2001). The reliability of angular measurements in hallux valgus deformities. Foot Ankle Int..

[34] Choi Y.R., Lee S.J., Kim J.H., Kim T.H., Oh C.H. (2018). Effect of metatarsal osteotomy and open lateral soft tissue procedure on sesamoid position: Radiological assessment. J. Orthop. Surg. Res..

[35] Huang E.H., Charlton T.P., Ajayi S., Thordarson D.B. (2013). Effect of various hallux valgus reconstruction on sesamoid location: A radiographic study. Foot Ankle Int..

[36] Easley M.E., Trnka H.J. (2007). Current concepts review: Hallux valgus part II: Operative treatment. Foot Ankle Int..

[37] Kilmartin T.E., O’Kane C. (2010). Combined rotation scarf and Akin osteotomies for hallux valgus: A patient focussed 9 year follow up of 50 patients. J. Foot Ankle Res..

[38] Lehman D.E. (2003). Salvage of complications of hallux valgus surgery. Foot Ankle Clin..

[39] Park C.H., Lee W.C. (2017). Recurrence of Hallux Valgus Can Be Predicted from Immediate Postoperative Non-Weight-Bearing Radiographs. J. Bone Jt. Surg. Am..

[40] Tanaka Y., Takakura Y., Kumai T., Sugimoto K., Taniguchi A., Hattori K. (2008). Proximal spherical metatarsal osteotomy for the foot with severe hallux valgus. Foot Ankle Int..

[41] Veri J.P., Pirani S.P., Claridge R. (2001). Crescentic proximal metatarsal osteotomy for moderate to severe hallux valgus: A mean 12.2 year follow-up study. Foot Ankle Int..

[42] Augoyard R., Largey A., Munoz M.A., Canovas F. (2013). Efficacy of first metatarsophalangeal joint lateral release in hallux valgus surgery. Orthop. Traumatol. Surg. Res..

[43] Schneider W. (2013). Distal soft tissue procedure in hallux valgus surgery: Biomechanical background and technique. Int. Orthop..

[44] Okuda R., Kinoshita M., Morikawa J., Yasuda T., Abe M. (2005). Proximal metatarsal osteotomy: Relation between 1- to greater than 3-years results. Clin. Orthop. Relat. Res..

